# Dental Ethics

**Published:** 1899-06

**Authors:** J. L. Young


### Dental Ethics.

BY DR. J. L. YOUNG, D.D.S.

Read before the Detroit Dental Society.

Ethics is the art of directing the actions of individuals for the benefit of society in general. Professional Ethics is the ethics of the professional classes. And thus it follows that Dental Ethics is the art of directing the actions of dental practitioners for the benefit of all society requiring dental services, as well as for the benefit of his fellow practitioners. Consequently the ethical rules of the society should embrace the actions of the members at society meetings, his conduct in his office, and his professional dealings in general.

The American Dental Association has adopted a code of ethics which, with slight modifications, has been adopted by nearly all local societies. Since we are supposed to be guided by it, let us examine it and determine if it covers the ground prescribed, and if it is sufficiently lucid to bear no misinterpretation. In order to be better able to do this I shall quote it as adopted that we may have it before our minds.

### CODE OF DENTAL ETHICS.

### ARTICLE I.

### The Duties of the Profession to their Patients.

*Section* 1.—The dentist should be ever ready to respond to the wants of his patrons, and should fully recognize the obligations involved in the discharge of his duties toward them. As they are, in most cases, unable to correctly estimate the character of his operations, his own sense of right must guarantee faithfulness in their performance. His manner should be firm, yet kind and sympathizing, so as to gain the respect and confidence of his patients; and even the simplest case submitted to his care should receive that attention which is due to any operation performed on living, sensitive tissue.

*Section* 2.—It is not to be expected that the patient will possess a very extended or a very accurate knowledge of professional matters. The dentist should make due allowance for this, patiently explaining many things which may seem quite clear to himself, thus endeavoring to educate the public mind so that it will properly appreciate the beneficent efforts of our profession. He should encourage no false hopes by promising success where, in the nature of the case, there is uncertainty.

*Section* 3.—The dentist should be temperate in all things, keeping both mind and body in the best possible health, that his patients may have the benefit of that clearness of judgment and skill which is their right.

ARTICLE II.

MAINTAINING PROFESSIONAL CHARACTER.

*Section* 1.—A member of the dental profession is bound to maintain its honor, and to labor earnestly to extend its sphere of usefulness. He should avoid everything in language and conduct calculated to discredit or dishonor his profession, and should ever manifest a due respect for his brethren. The young should show special respect to their seniors; the aged special encouragement to their juniors.

*Section* 2.—The person and office arrangements of the dentist should indicate that he is a gentleman; and he should sustain a high-toned moral character.

*Section* 3.—It is unprofessional to resort to public advertisements, such as cards, hand-bills, posters, or signs calling attention to peculiar styles of work, prices for services, special modes of operating, or to claim superiority over neighboring practitioners; to publish reports of cases or certificates in the public prints; to go from house to house soliciting or performing operations; to circulate or recommend nostrums, or to perform any other similar acts. But nothing in this section shall be so construed as to imply that it is unprofessional for dentists to announce in the public prints, or by card, simply their names, occupation, and place of business; or in the same manner, to announce their removal, absence from, or return to business; or to issue to their patients, appointment cards having a fee-bill for professional services thereon.

*Section* 4.—When consulted by the patient of another practitioner the dentist should guard against inquiries or hints disparaging to the family dentist, or calculated to weaken the patient's confidence in him; and if the interests of the patient will not be endangered thereby, the case should be temporarily treated and referred back to the family dentist.

*Section* 5.—When general rules shall have been adopted by members of the profession practising in the same localities, in relation to fees, it is unprofessional and dishonorable to depart from these rules, except when variation of circumstances requires it. And it is ever to be regarded as unprofessional to warrant operations or work as an inducement to patronage.

ARTICLE III.

THE RELATIVE DUTIES OF DENTISTS AND PHYSICIANS.

Dental surgery is a specialty in medical science. Physicians and dentists should both bear this in mind. The dentist is professionally limited to diseases of the dental organs and the mouth. With these he should be more familar than the general practitioner is expected to be; and, while he recognizes the superiority of the physician in regard to diseases of the general system, the latter is under equal obligation to respect his higher attainments in his specialty. Where this principle governs there can be no conflict, or even diversity, of professional interests.

ARTICLE IV.

MUTUAL DUTIES OF THE PROFESSION AND THE PUBLIC.

Dentists are frequent witnesses, and at the same time the best judges, of the impositions perpetrated by quacks, and it is their duty to enlighten and warn the public in regard to them. For this, and the many other benefits conferred by the competent and honorable dentist, the profession is entitled to the confidence and respect of the public, who should always discriminate in favor of the true man of science and integrity, and against the empiric and impostor. The public has no right to tax the time and talents of the profession in examinations, prescriptions, or in any way without proper remuneration.

In Article II, Section 1, "A member of the dental profession is bound to maintain its honor and to labor earnestly to extend its sphere of usefulness." First let us consider what is necessary to become a member of the dental profession. In this State all that is necessary is to pass an examination before the State Board, while in other States a diploma from a recognized college and registration are required. In either case what is there to compel one so educated to maintain the honor of the profession? Can the State Board revoke the certificate, or the college demand the return of the diploma granted and paid for? If this cannot be done, and if it can it is not, how can a society enforce this, having no jurisdiction over members of the profession until they become members of the society?

Again, in the same section it would seem that the following would be better left out, "The young should show special respect to their seniors; the aged special encouragement to their juniors." If dentistry is to stand as a scientific profession and our colleges do their duty, then it follows that a graduate who joins the society must be worthy of the same respect and consideration as the aged practitioner. While it is not to be supposed that all the members of a society are possessed of the same ability, yet, I claim it is the duty of the society to show equal respect to individual members and their opinions on all matters brought before the society. And each member should respect the society enough to be willing to do his share towards advancing interests, and when down for a paper or to lead in the discussion of a paper, he should be punctual and prepared. And each member should respect the opinion of every other member of the society. And, as well, in every-day practice each should be ever careful of criticising, either by word or act, operations performed by another.

In Section 3 of the same article we find the bugbear of ethics, advertising. Let us first consider the clause, "It is unprofessional to claim superiority over neighboring practitioners." Does this refer to the glaring advertisements of the quack, who claims he is the only practitioner performing certain operations, or does it also prohibit the gentleman who is working up a legitimate practice to hold out inducements to those whose teeth have been improperly filled by his neighboring practitioner? To make this plain we will suppose a case. A lady applies to Dr. A to have her teeth extracted, they having been filled by Dr. B a few months previous and are now in bad condition. Dr. A finds by careful examination that the operations were poorly performed, and feels sure he can save the teeth indefinitely. Is it wrong, if necessary to convince the lady of his ability to do so, for Dr. A to say that Dr. B has not properly performed the operations? Or must he, in order to be ethical, extract her teeth and supply the artificial substitutes asked for?

Another clause in this same section says, "It is unprofessional to circulate or recommend nostrums." A nostrum is a medicine the composition of which is kept secret. I will leave it for yourselves to answer how many live up to the code in this particular. There are many other things which in spirit are just as objectionable as any of those mentioned in this section. For instance, having different grades of amalgam fillings made from the one alloy; or different priced rubber dentures; or to have a photograph and biography in a special edition of the newspaper; or to send the reporter a receipted bill for professional services, in order that he may publish a flattering description of your office appointments.

Section 4 of the same article does not define who is to be regarded as a patient of another practitioner. Is it to be inferred that any one having work recently done should be referred back to the original operator if the operations have been satisfactory? Or does it refer to those suffering from some unexpected cause, and who are dissatisfied with the operator and have decided to try another? Or does it refer to those who are in pain, and, owing to the absence of the family dentist, have sought relief elsewhere? In my opinion the last mentioned are those only to whom this should apply.

Again I have a very serious objection to that part of Section 5 which says, "It is unprofessional and dishonorable to depart from rules relating to fees of practitioners in the same locality." I contend it is unprofessional for dentists in the same locality to enter into such an agreement, for is this not benefiting the practitioner at the expense of society? It also works a hardship on the younger practitioners, who are bound to demand the same fees as their seniors with established clientele. It would be a very good way to drive the young fellows somewhere else.

Besides the written code we have the unwritten or broader one, founded on the moral law "Do unto others as you would wish others to do unto you." Some claim that the unwritten code is sufficient to govern the actions of a gentleman, and those who will not be guided by it cannot be constrained by the written code. This may be true, but it is necessary for societies to have some rules for membership and these should be in the spirit of the unwritten code, strictly in accordance with the actions of a gentleman. They should be clear, so that the unprincipled could not act in an unprofessional way and still obey the letter of the code, and should be so full that there would be no loophole for dishonorable dealings. Had we such an ideal code, it would tend to the elevation of the society members, thus to the elevation of the society and to the elevation of the whole profession.

### DISCUSSION ON DR. YOUNG'S PAPER ON DENTAL ETHICS.

BY DR. M. E. STAFFORD.

The essayist, in criticising the Code of Ethics, Article II, Section 1, which treats of the duty of a member of the profession to maintain its honor, etc., etc., says, "Our State allows any one to become a member of the profession on passing an examination before the State Board" * * and "In either case what is there to compel one so educated to maintain the honor of the profession?" He asks how the society can enforce this having no jurisdiction, etc. The place, in my opinion, for the society to get its work in, and one of its objects and duties, is to encourage proper legislation and to instigate and aid by its united influence the passage and enforcement of such laws in this State and other States as shall enable a college to nullify a diploma or a State Board to revoke a certificate, and to make it dangerous to practice without these qualifications. And why not? Let a teacher of the public schools conduct himself unbecomingly and see how quickly his certificate will be revoked or annulled, from whatever source he obtained it, after which he can draw no salary for his services until he is reinstated.

Our essayist seems to have a special grudge against Article II, Section 1, for he says that the following clause would be better left out, viz.: "The young should show special respect to their seniors; the aged, special encouragement to their juniors." I might agree with him in part, inasmuch as this would only enjoin upon a member, common decency or the gentlemanly behavior which is elsewhere prescribed in the code, but I should wish to transplant this clause to the unwritten code he speaks of further on in his paper.

It might be well to insert the words "in practice" after "seniors" and "juniors," making the clause to read, "The young should show special respect to their seniors in practice," etc. This I think is the idea intended in the code, as I know it would not be desirable for the essayist to show me any special respect; for while I may be his senior in years I am not so in practice, hence the respect then would devolve upon me to show to him and the active part in the encouragement plan would fall to him. I think we have both unconsciously lived up to the code in this way.

In any event, I think the aged practitioner is *entitled* to special respect, for while he may not know any more than the average graduate thinks he knows, still I think our essayist must concede him that superiority in skill which is only acquired in the doing many, many times over, the myriad of operations in the practice of dentistry requiring delicate manipulative ability.

Are we to understand the essayist that his own opinion is entitled to no more respect or consideration now than years ago when he first graduated? I must beg to differ with him there, except perhaps on one subject, the one under discussion, in which he does not seem to have progressed as rapidly as in some others. A man must be nonprogressive indeed who does not improve and increase his knowledge in years of practice so as to entitle him to more consideration and respect for his ideas than when he first cracked his college shell.

Again, in the case supposed by the essayist in which Dr. B's patient comes to Dr. A to have teeth extracted which were filled by Dr. B only a few months previously. Dr. A finds they were not properly filled and feels sure he can save the teeth indefinitely. He asks, "Is it wrong for Dr. A to say that Dr. B has not properly performed the operations? Or must he, in order to be ethical, extract her teeth and supply the artificial substitutes asked for?" In the first place, the patient probably realizes that Dr. B's work is not what it should be else why does she not go to him to have them extracted? It is evident that she has lost confidence in Dr. B, and through his lack of skill or carelessness he has lost his patient; but it is not necessary for Dr. A

to tell her that Dr. B has not properly performed his work, if she has average intelligence she knows that, nor does it in any way prove Dr. A's ability to do the work properly. Let the poor fellow down as easily as possible, but don't jump on him. To quote the language of the essayist himself, on a previous page, he says, "And, as well, in every-day practice each should be ever careful of criticising, either by word or act, operations performed by another." That could not have referred to Dr. A but the "*other fellow.*" Or does the item of dollars and cents come into the reckoning? Then he should word it, "criticise only when there is money in it." There might have been extenuating circumstances on Dr. B's side, perhaps the patient's behavior precluded the best work; don't criticise Dr. B's work until you have done better, and then let your work condemn him rather than your tongue. Dr. A might say, perhaps, "Dr. B did not do the work just as I should have done it," then if she gives him the chance he can show her the way and let her judge which is the best. "Or must he," asks the essayist, "in order to be ethical, extract her teeth and supply the artificial substitutes asked for?" That, to my notion, would be the most unethical course he could pursue. If he was sure the teeth ought not to be extracted, and failed to induce her by fair argument and explanation to allow him to fill them, then the code, it seems to me, would have him refuse to extract them, having always the patient's best interests at heart.

